# Identification of a novel SPT inhibitor WXP-003 by docking-based virtual screening and investigation of its anti-fungi effect

**DOI:** 10.1080/14756366.2021.1915301

**Published:** 2021-06-21

**Authors:** Xin Wang, Xin Yang, Xin Sun, Yi Qian, Mengyao Fan, Zhehao Zhang, Kaiyuan Deng, Zaixiang Lou, Zejun Pei, Jingyu Zhu

**Affiliations:** a The Affiliated Wuxi No. 2 People’s Hospital of Nanjing Medical University, Wuxi, China; b School of Food Science and Technology, Jiangnan University, Wuxi, China; c School of Pharmaceutical Sciences, Jiangnan University, Wuxi, China

**Keywords:** Virtual screening, SPT, inhibitor, anti-fungi

## Abstract

Serine palmitoyltransferase (SPT) plays the key role on catalysing the formation of 3-ketodihydrosphingosine, which is the first step of the *de novo* biosynthesis of sphingolipids. SPT is linked to many diseases including fungal infection, making it a potential therapeutic target. Thus, a logical docking-based virtual screening method was used to screen selective SPT inhibitor against fungi, not human. We used myriocin-similarity database to identify compounds with good binding with fungal SPT and poor binding with homology human SPT model. Preliminary bio-assay led to the discovery of a promising inhibitor **WXP-003**, which displayed good inhibitory activity against diversity fungi strains with MIC ranging from 0.78 to 12.5 μg/mL. **WXP-003** could significantly reduce sphingolipids content in fungi and no effect on mouse fibroblast cell line L929. Molecular dynamics simulation depicted that SPT/**WXP-003** complex formed the favoured interactions. Taken together, discovery of **WXP-003** provided valuable guide for the development of novel anti-fungal agents.

## 1. Introduction

Fungal infection is one of main infectious diseases in clinic, including common superficial and invasive fungal infection^1^. In the past few decades, morbidity and mortality caused by invasive fungal infection have been increasing with the sharp growing immunocompromised individuals, such as patients after organ transplant, patients in ICU (morbidity up to 29%, mortality up to 49%)^2^^,^^3^. Due to the increasing life-threatening caused by fungal infection, the effective treatments for fungal disease are needed. However, the approved antifungal agents are quite limited, mainly containing polyenes (e.g. amphotericin B and its derivatives), azoles (e.g. fluconazole, ketoconazole), echinocandins (e.g. micafungin) and 5-fluorocytosine^4^. With the extensive use of conventional antifungal agents, the emerging azole-resistant fungi make the problem more intractable^5–7^. Moreover, the current antifungal agents always show low efficacy on killing fungal cells and high toxicity because of the similarities between fungi and mammals. Therefore, developing novel antifungal agents against new targets is urgent need to improve the efficacy in killing fungi and decrease the side effects^8^.

Viewed as important structural components of cellular membranes, sphingolipids are a large class of lipids which implicate a wide range of cellular functions in eukaryotic and prokaryotic organisms^9–12^. Except that, sphingolipids play other multiple roles in fungal cells specifically, such as heat stress response, signal transduction, endocytosis, apoptosis and many others. In particular, it is also one of causes of fungal pathogenesis^13–15^. Thus, highly concerning on the biology of sphingolipids would be helpful for searching new targets against fungal infection. *De novo* sphingolipids biosynthesis is a complicated process mediated by a large number of enzymes^16^. Serine palmitoyltransferase (SPT) is a heterodimeric membrane protein, that could catalyse the formation of 3-ketodihydrosphingosine (3-KDS) from serine and palmitoyl CoA through condensation reaction^17^. SPT is the initial and rate-limiting enzyme in the *de novo* biosynthesis of sphingolipids, directly affecting the synthesis of two main groups of sphingolipids in fungal cells, such as inositol phosphoryl ceramide (IPC) and glucosylceramide (GlcCer)^11^^,^^18–20^. Some studies have shown that inhibiting the enzymes involved in the biosynthetic pathways of sphingolipids could decrease the virulence of fungal pathogens^21^. Especially, SPT inhibitors have been reported that could inhibit the sphingolipid formation effectively, resulting in changes of fungal membranes and cell morphology. Therefore, SPT could be an attractive target against fungal infection^13^.

**Figure 1.**
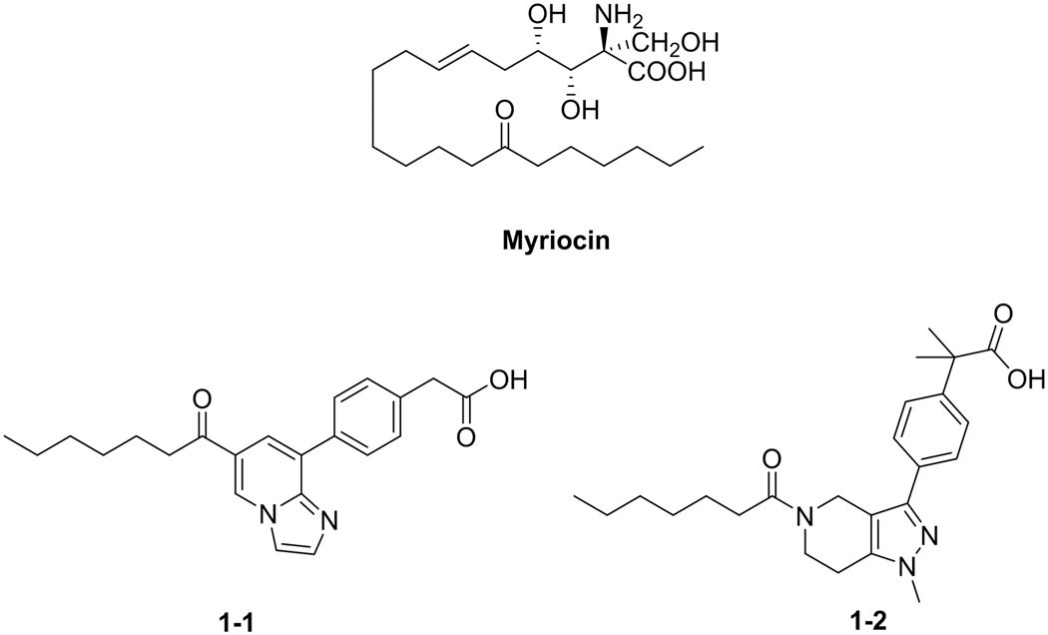
The structures of reported SPT inhibitors.

SPT inhibitors have been reported include myriocin and some synthesised compounds **1–1**∼**2** (Figure 1), and one of the most studied on anti-fungi SPT inhibitor is myriocin with good inhibitory efficacy against yeast and mould in planktonic and effecting formation of biofilm. However, SPT is also existed in host cell, leading toxicity to mammalian after administration. As well as, there are few reports on selective SPT inhibitor to date^22^^,^^23^. Thus, it is highly desirable to find SPT inhibitor specifically targeting fungi, rather than mammalian.

**Figure 2.**
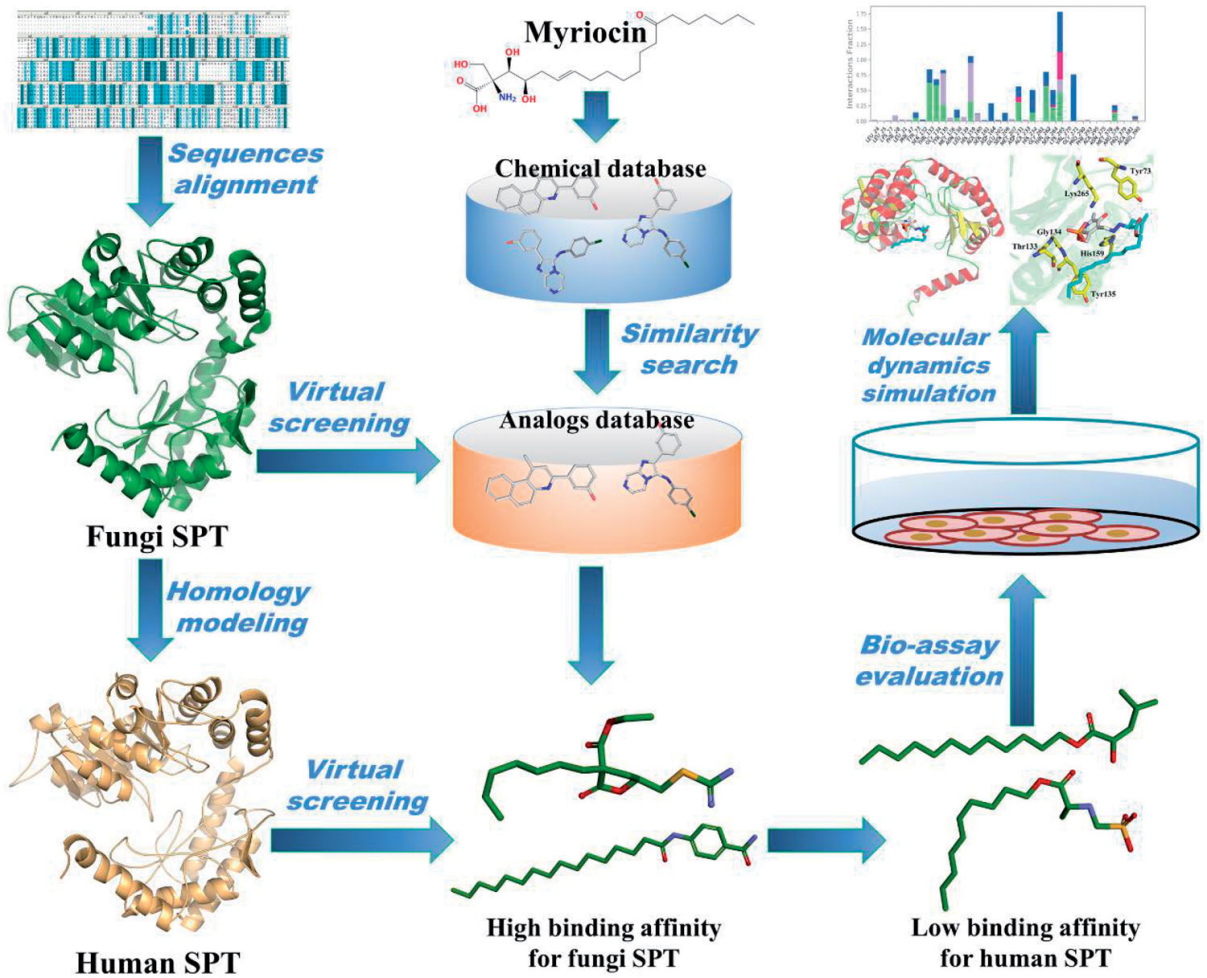
The workflow of this study.

Since there is no 3D structure of human SPT-inhibitor complex reported at present, we finished homology human SPT model based on fungi SPT and assessed the quality of the homology SPT model through the Ramachandran plot generated by SAVES server. In effort to discover selective SPT inhibitor against fungi, we performed docking-based virtual screening (VS). Based on myriocin is a well-known SPT inhibitor, we hypothesised myriocin-analogues might have higher chances on good binding with SPT^24^. At the beginning, myriocin-similarity database was applied to molecular docking with fungi SPT and human SPT in turn. Based on further biological evaluation, we discovered a potent and novel SPT inhibitor with good inhibitory activity against diversity fungi. This study provided a valuable lead for development of higher potent and lower toxic SPT inhibitor. The screening procedure is summarised in Figure 2.

## 2. Materials and methods

### 2.1. Homology modelling

The protein sequence of human SPT (UniProtKB: O15269) was retrieved from the NCBI database (https://www.ncbi.nlm.nih.gov/protein/). BLAST search(BLASTP) (https://blast.ncbi.nlm.nih.gov/Blast.cgi) was employed to search the template proteins for building human SPT, the search database was set to protein data bank (PDB), and the other parameters used defaults. After sequences search, four proteins structures were chosen, namely, 2X8U (PDB ID)^25^, 2JG2^26^, 3A2B^27^ and 4BMK^11^, for the following homology modelling (HM). First, the *Align Sequence to Templates* module of Discovery Studio 3.5 (DS3.5) was employed to align the human SPT sequence and the template protein sequences. Second, the *Build Homology Models* module in DS3.5 was used to construct the target protein with the above sequence alignment, and the parameters were set as default. The top-scored model was chosen and then submitted to verify using the SAVES v5.0 server (https://servicesn.mbi.ucla.edu/SAVES/).

### 2.2. Similarity search

The 3D structure of myriocin was retrieved from the Pubchem database (PubChem CID: 6438394, https://pubchem.ncbi.nlm.nih.gov/). The Specs and Chemdiv databases were prepared by the *Prepare Ligands* protocol in DS3.5. And then, the *Find Similar Molecules by Fingerprints* module was employed with the Tanimoto coefficient^28^, myriocin was used as a search query molecule, and 10 fingerprint methods were used, including three FCFPs (FCFP_2, FCFP_4, and FCFP_6), three ECFPs (ECFP_2, ECFP_4 and ECFP_6), three FCFCs (FCFC_2, FCFC_4, FCFC_6), and one MDL Public Keys.

### 2.3. Virtual screening (VS)

*Preparation of protein*: The crystallographic structure of SPT (PDB ID: 4BMK, *Sphingomonas paucimobilis*) was used as the initial receptor of subsequent molecular docking virtual screening. The protein was prepared using the *Prepare Protein protocol* in DS3.5, to remove all crystallographic water, add hydrogen atoms, repair broken chains, and add CHARMm force field. Afterward, a sphere binding site was generated with the co-crystallized myriocin as centroid using the define site tool in DS3.5.

*Molecular docking*: The CDOCKER protocol in DS3.5 was used to perform the VS. First, the prepared 4BMK was submitted to screen the similarity molecules, the top hits were set to 5 and the other parameters were set as default. Afterward, the top-scored pose of each similarity molecule was retained, and all selected docking poses were submitted to VS with the modelling-human SPT protein.

### 2.4. Chemistry

*The synthesis of compound* **WXP-001**: l-Serine (**1**, 2.0 g, 19.0 mmol) was dissolved in 80 ml of toluene, and dodecanol (**2**, 3.5 g, 19.0 mmol), *p*-toluenesulfonic acid monohydrate (3.6 g, 19.0 mmol) in turn were added to above solution at room temperature. Then the oil–water separator was connected and heated to 110 °C under reflux overnight. The reaction was monitored by TLC. The reaction solution was cooled to room temperature and diluted with ethyl acetate. Then the organic phase was washed with saturated sodium bicarbonate and brine successively and dried with anhydrous sodium sulphate. After filtered, the filtrate was concentrated *in vacuo* through a silica gel column (PE:EA = 1:1–1:3∼DCM:MeOH = 20:1) to obtain 2.5 g of brown oil l-serine lauryl ester. The resultant brown oil (800 mg) was dissolved in 5 ml mixture solution containing hydrogen chloride and methanol, stirring at room temperature for 15 min, and concentrated under vacuum to obtain the product l-serine dodecyl ester hydrochloride (compound **WXP-001**, 410.0 mg, 31%), light brown particles. LC-MS(ESI): 274.4 (M + H)^+^, RT = 4.75 min; ^1^H NMR (400 MHz, DMSO-d_6_) *δ* 8.43 (s, 2H), 5.59 (s, 1H), 4.19–4.11 (m, 3H), 3.86–3.77 (m, 2H), 1.64–1.57 (m, 2H), 1.32–1.25 (m, 18H), 0.88–0.84 (m, 3H).

*The synthesis of compound* **WXP-002**: l-Leucine (**3**, 1.31 g, 0.01 mol) was dissolved in toluene (20 ml), then added octadecan-1-ol (**4**, 2.70 g, 0.01 mol) and *p*-toluenesulfonic acid (2.09 g, 0.01 mol) to above solution. Use the method of synthesis of **WXP-001**. Purified through silica gel chromatography (PE:EA = 2:1) to give yellow oily leucine octadecy ester (compound **WXP-002**, 2.21 g, 57%). LC-MS(ESI): 384.5 (M + H)^+^, RT = 6.68 min; ^1^H NMR (400 MHz, DMSO-d_6_) *δ* 8.48 (s, 2H), 4.20–4.09 (m, 2H), 3.97–3.93 (m, 1H), 1.76–1.72 (m, 1H), 1.64–1.58 (m, 4H), 1.29–1.23 (m, 30H), 0.91–0.89 (m, 6H), 0.87–0.83 (m, 3H).

*The synthesis of compound* **WXP-003**: l-Leucine (**3**, 1.31 g, 0.01 mol) was dissolved in toluene (20 ml), then added dodecanol (**2**, 1.68 g, 0.01 mol) and *p*-toluenesulfonic acid (2.09 g, 0.01 mol) to above solution. Use the method of synthesis of **WXP-001**. Purified through silica gel chromatography (PE:EA = 2:1) to give yellow oily leucine dodecyl ester (1.0 g, 33%). LC-MS(ESI): 300.3 (M + H)^+^, RT = 2.94 min; ^1^H NMR (400 MHz, CDCl_3_) *δ* 4.12 (t, 2H), 3.46 (m, 1H), 1.83 (m, 2H), 1.77–1.75 (m, 1H), 1.74–1.72 (m, 2H), 1.45–1.20 (m, 18H), 0.95–0.83 (m, 9H).

### 2.5. MIC determination via broth microdilution assay

MIC determinations were finished via broth microdilution assay with myriocin and caspofungin used as positive inhibitor controls. All experimental fungus were provided by the Pathogenic Microorganism (toxin) Preservation Center of Chinese Academy of Medical Sciences, including four species of fungi and two drug-sensitive reference strains as follows: *Aspergillus fumigates* (CMCCC(F) A.1a), *Cryptococcus neoformans* (CMCCC(F) D.2a), *Sporothrix* (CMCCC(F) D.1a), *Rhizopus oryzae* (CMCCC(F) B.81a), *Candida parapsilosis* (ATCC22019) and *Candida krusei* (ATCC6258). Myriocin was prepared to 2 mg/mL solution in methanol and caspofungin was prepared to 5 mg/mL solution in water. Compounds **WXP-001∼003** were dissolved in dimethyl sulfoxide (DMSO) to prepare stock solutions of 10 mg/mL concentration.

The compounds along with positive controls were serially diluted to 10 concentrations solution across the 96-well plates, ranging from 100 µg/mL to 0.195 µg/mL with final volumes of 100 µL per well. Yeasts were cultured in Sabouraud Dextrose Agar (SDA) at 30 °C for 48 h. Filamentous fungus were cultured in Potato Dextrose Agar (PDA) at 26 °C for 7–10 d. Each sample was then diluted to suspension with sterile saline solution and was calculated the density. The resultant suspensions were diluted and added to above compound-containing 96-well plates (100 µL per well) to give the yeasts density of 0.5–2.5 × 10^3^ cfµ/mL and filamentous fungus density of 0.4–5 × 10^4^ cfµ/mL. All the plates were incubated at 37 °C. The OD value of each well was recorded, the blank subtracted out, and the growth in each well calculated as percentage of the control. The lowest concentration of compound that resulted in <10% growth was recorded as the MIC.

### 2.6. Sphingolipids content assay

Cells in the logarithmic phase were removed to a centrifuge tube and sonicated for 30 min in an ice bath. Fungi were also treated under same conditions. Collected 1.0 ml of supernatant by centrifugation, then added 2.0 mL of extraction mixture (isopropanol: ultrapure water: ethyl acetate = 30:10:60). The mixture was extracted for 2 h at 180 rpm and separated by standing or centrifugation, and the supernatant was taken as the test sample. Diluted the standard and determined the standard curve. Set blank well and sample well, added 40 µL of sample diluent to the 96-well plates, and added 10 µL of sample to be tested (the final dilution concentration of the sample is 5 times). After sealed with the sealing film, the plates were incubated at 37 °C for 30 min. Diluted the 30-fold concentrated solution with distilled water 30-fold before use. Then each well was washed with washing liquid for 5 times, each time for 30 s. Except for blank wells, sample wells were added 50 µL of enzyme-labelled reagent. Repeated the incubation operation and washed. Each well was added a colour developer and mixed gently, then incubated at 37 °C for 10 min in the dark. After incubation, added 50 µL stop solution to stop the reaction. OD value was measured at 450 nm.

### 2.7. Molecular dynamics (MD) simulation

The SPT/**WXP-003** complex, with the best bioactivity, generated by molecular docking was used as initial structures to carry out MD simulation by *Desmond* software package^29^. First, the whole system was immersed in a solvent orthorhombic model with explicit SPC (Single Point Charge) water molecules and the boundary of the infiltration volume was extended to 10 Å in each direction, and then the system was neutralised with Na^+^/Cl^−^ counter ions. Further, the solvated system was minimised under the OPLS_2005 force field. Afterward, the complex was submitted to a 60-ns MD simulation following the default NPT and NVT ensemble protocols in *Desmond* program. During the production run, the temperature and the pressure were set to 300.0 K and 1.01325 bar, respectively. The energy and atomic coordinate trajectory recording interval were set to 20 ps. After MD simulation, The RMSD and protein-ligand contacts were calculated with the *Simulation Interaction Diagram* protocol in *Desmond* package^30^.

## 3. Results and discussion

### 3.1. In silico screening

The procedure of *in silico* screening employed in this study involves two stages of establishment of homology modelling and docking-based virtual screening.

**Figure 3.**
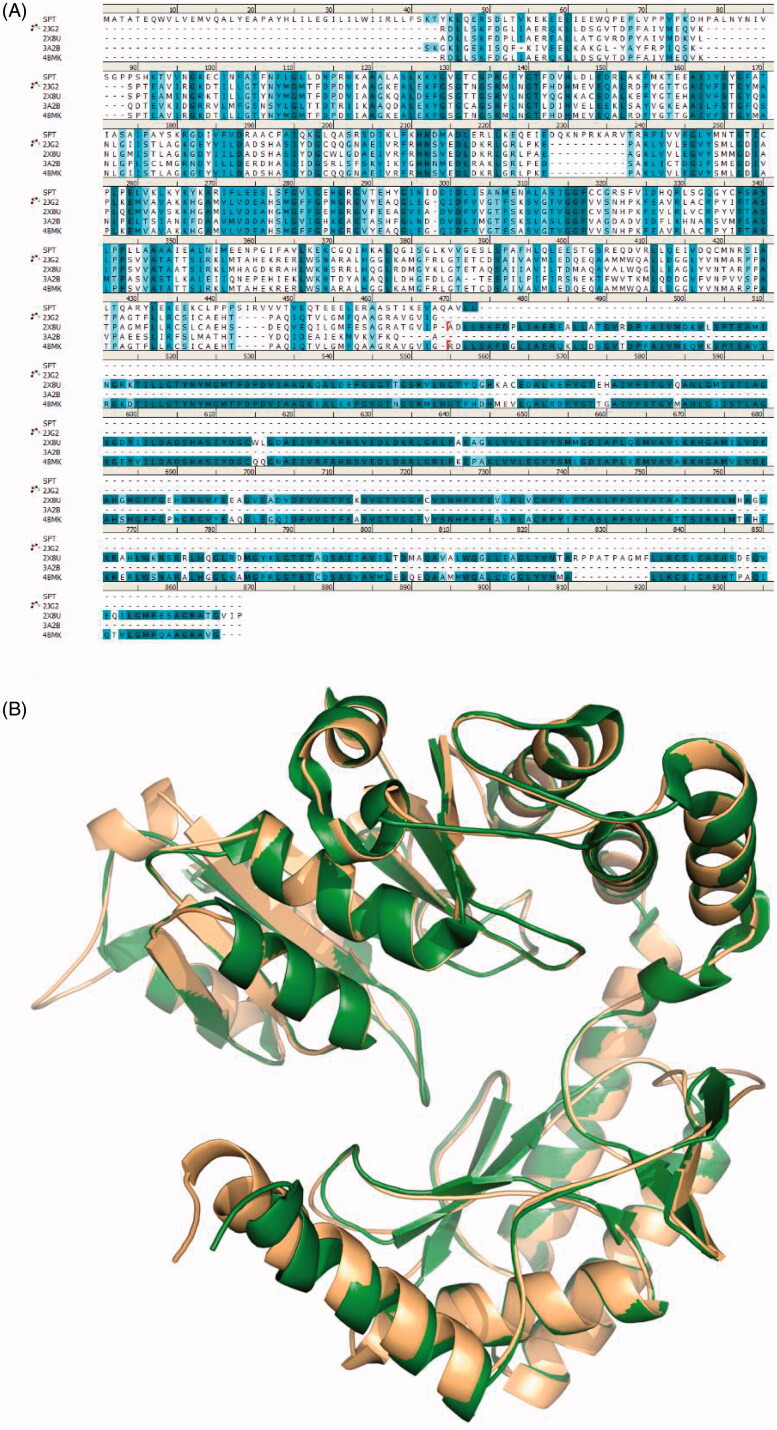
(A) The alignment of SPT sequences. (B) Alignment of homology SPT model (orange) and fungi SPT (green, PDB ID: 4BMK).

**Figure 4.**
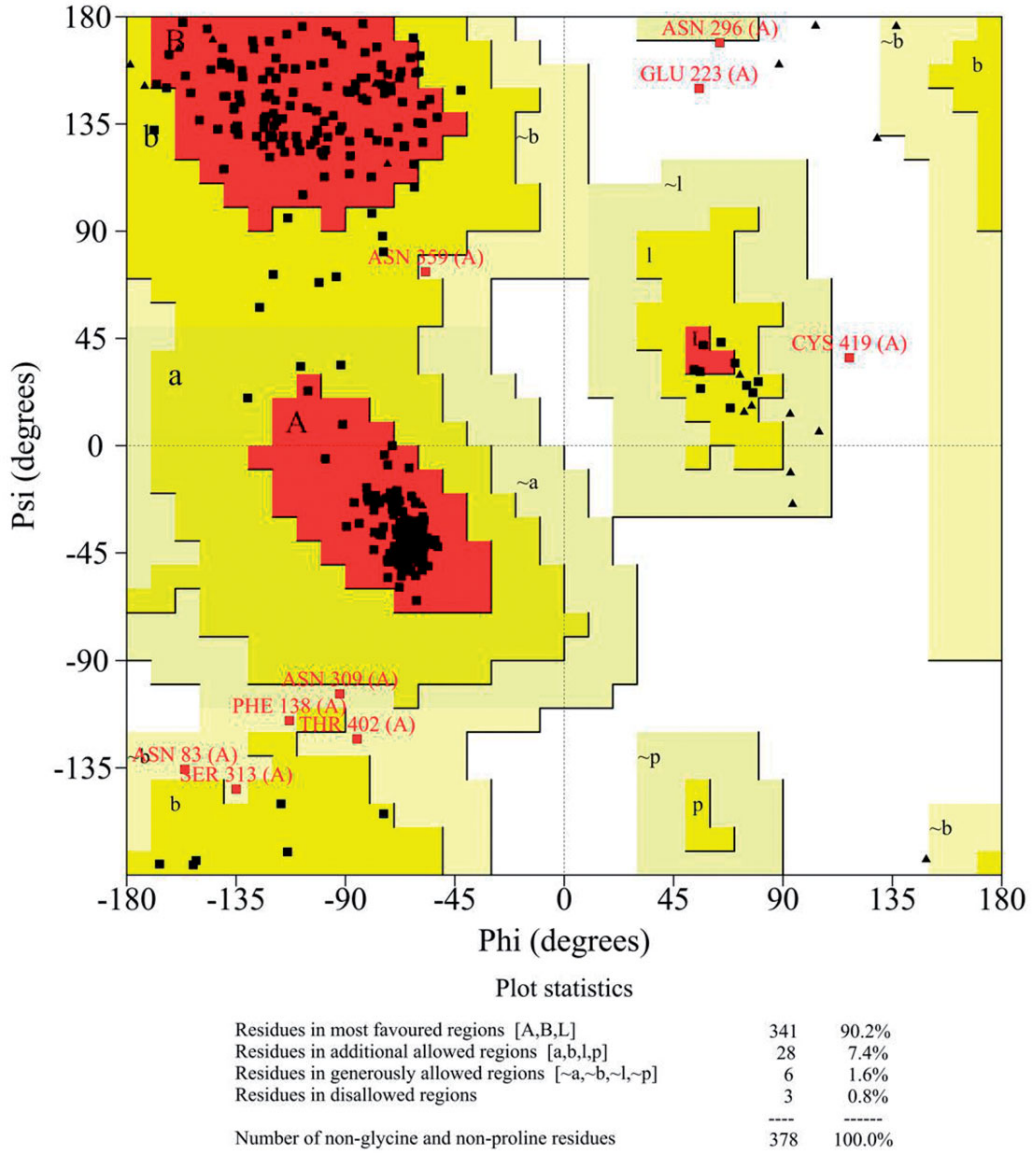
Ramachandran plot of homology SPT model.

Initially, the input human SPT sequences were searched to find homologous protein structures using BLAST, and this resulted four proteins as matching proteins in the PDB, structure ID 2X8U (a crystal structure of Sphingomonas *wittichii*), 2JG2 (a crystal structure of Sphingomonas *paucimobilis*), 3A2B (a crystal structure of Sphingobacterium *multivorum*) and 4BMK (a crystal structure of Sphingomonas *paucimobilis*). As is well known, the sequence alignment plays a key role in homology modelling method. From the BLASTP results, the human SPT exhibited a good alignment with the selected template proteins. The sequence identities were 29.41% for 2X8U, 28.44% for 2JG2, 32.33% for 3A2B, and 28.44% for 4BMK, and the alignments of structures and sequences showed in Figure 3. The quality of the homology SPT model was assessed through the Ramachandran plot generated by SAVES server. As shown in Figure 4, 90.2% amino acids were in most favoured regions, 7.4% amino acids were in additional allowed regions and only 0.8% were in disallowed regions. These results indicated that the building human SPT model was satisfactory and reliable for the following VS.

**Figure 5.**
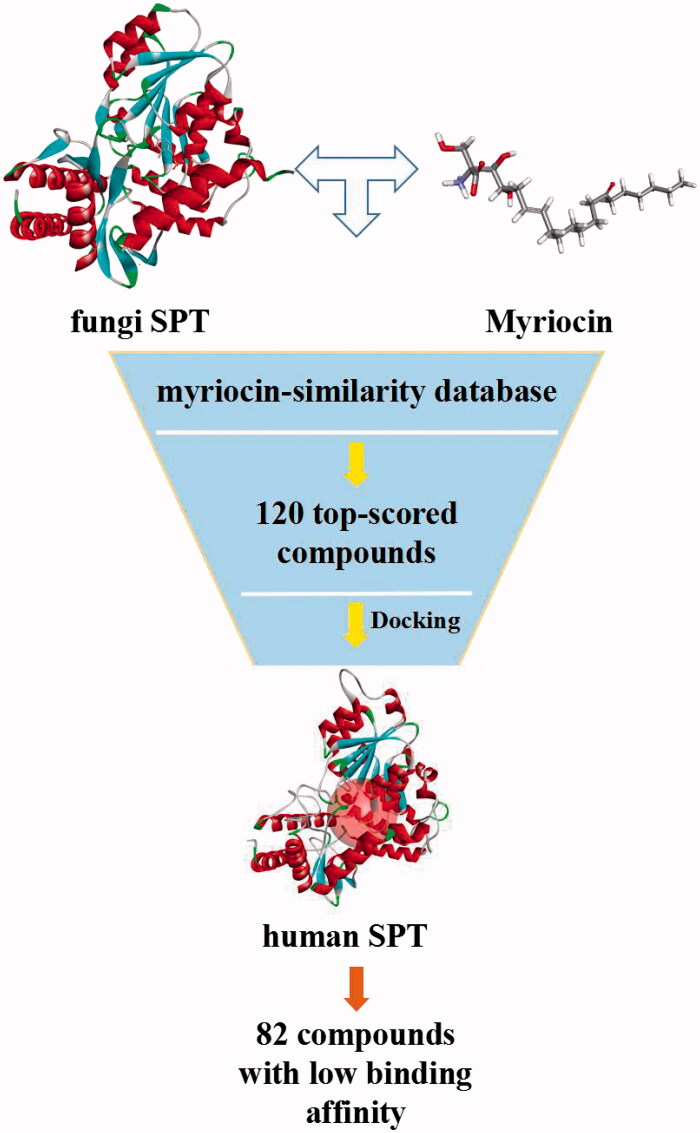
The procedure of virtual screening.

The procedure of virtual screening is shown in Figure 5. Since myriocin is a well-known SPT inhibitor, myriocin-analogues may have higher chances on good binding with SPT. Based on 3D structure of myriocin, the Specs and Chemdiv databases were systematic fingerprint to establish myriocin-similarity database. Then, the crystallographic structure of fungal SPT with myriocin (PDB ID: 4BMK) was employed to screen against the myriocin-similarity database. About 120 top-scored compounds were retained, and then were submitted to the homology human SPT model. Finally, 82 compounds with weak binding affinity to human SPT were selected for the further bio-assay.

### 3.2. Anti-fungal activity evaluation and chemistry

**Figure 6.**
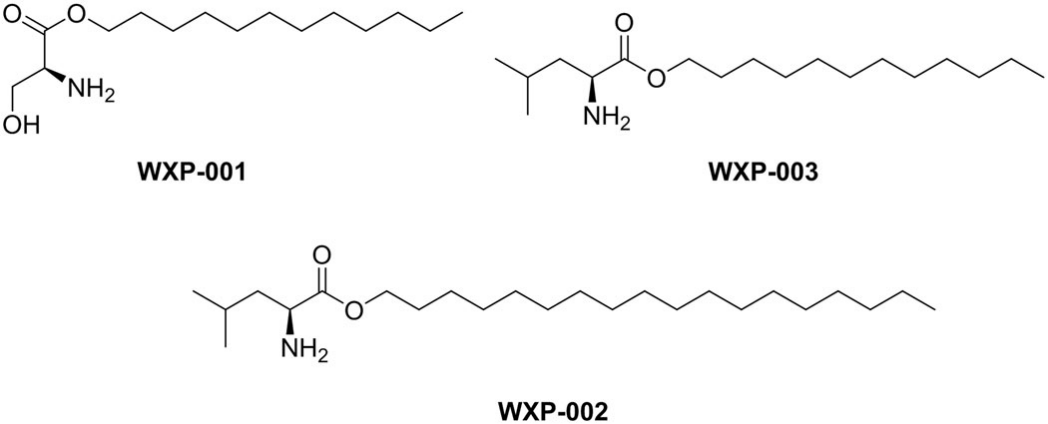
The structures of WXP-001∼003.

The 82 compounds screened *in silico* were evaluated through preliminary bio-assay. Five standard fungal strains (*C. tropicalis* 186815, *C. parapsilosis* 336515, *C. glabrata* 337348, *C. krusei* 185429 and *C. lusitaniae* 340928) and three clinical drug-resistance fungal strains (*C. tropicalis* 191529, *C. tropicalis* 191327 and *C. parapsilosis* 191344) were used as tested fungi. These compounds were initially screened to inhibit fungi growing at the concentration of 100 µM, 50 µM and 10 µM. Only 5 compounds displayed promising anti-fungal growing activity against diversity fungi at tested concentration (Supplementary Table S1). **WXP-004∼005** only showed low activity against some kinds of fungi. Especially, compounds **WXP-001∼003** showed significant anti-fungal activity against the tested fungi, the structures as shown in Figure 6. **WXP-001∼002** inhibited all the kinds of tested fungi growing at the concentration of 50 µM, while **WXP-003** revealed the brilliant inhibitory activity against all the tested fungal strains at 10* µ*M (Supplementary Table S1). Therefore, **WXP-001∼003** were selected to further evaluated on more kinds of fungi to detect the MIC value in detail.

**Scheme 1.**
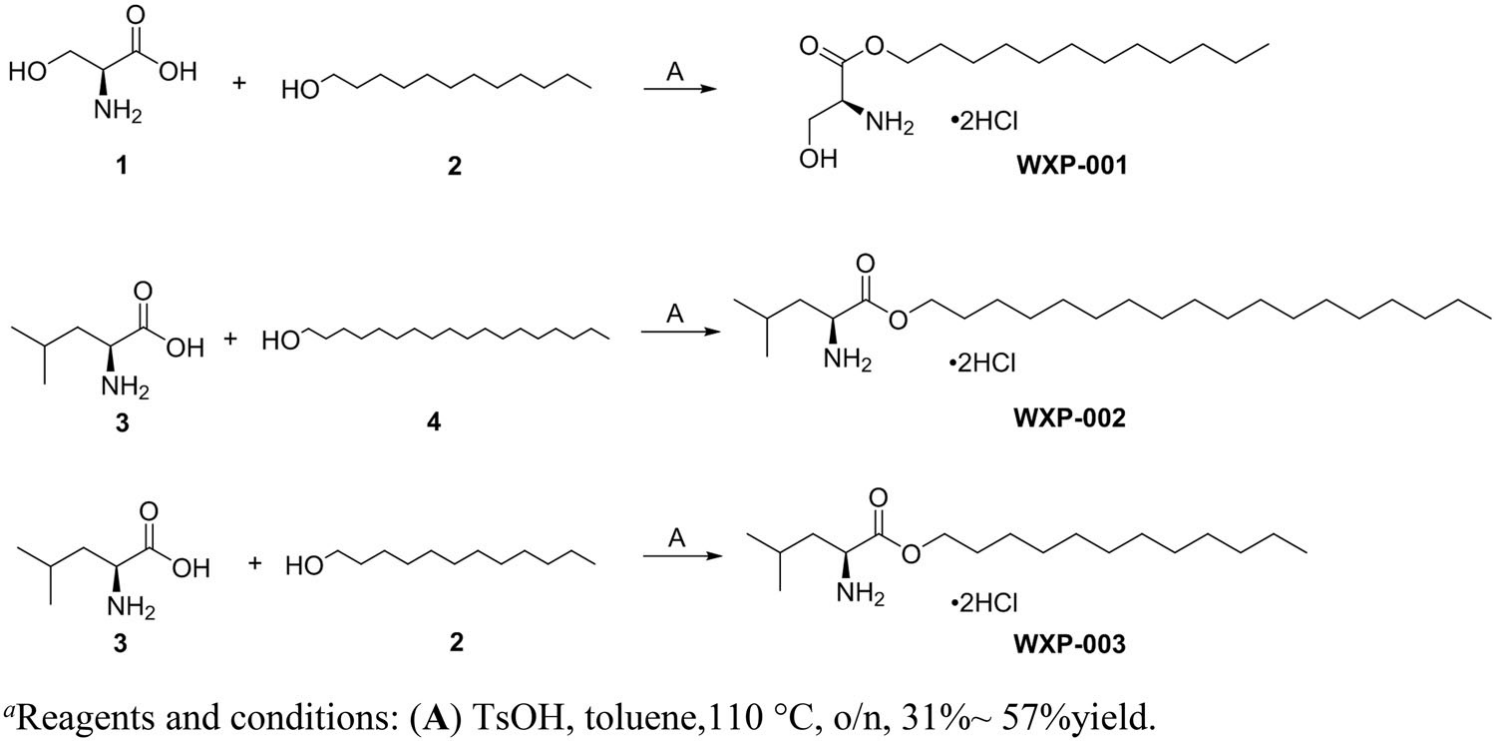
The synthesis of compound WXP-0 0 1–003. ^a^(Reagents and conditions: (**A**) TsOH, toluene,110 °C, o/n, 31%∼ 57%yield).

Based on above preliminary screening on anti-fungal activity, we first synthesised the three potential compounds to make sure enough amounts of compounds for the further evaluation. As shown in Scheme 1, compound **WXP-001** was synthesised through ester condensation reaction with l-serine and dodecanol as the start materials. Compounds **WXP-002∼003** were employed the same method of **WXP-001**. Notably, all the products were needed to be stirred in mixture solution of hydrogen chloride and methanol to produce hydrochloride product. All the steps obtained moderate yields.

Here, compounds **WXP-001∼003** exhibited fungicidal activity against six fungal strains evaluated in the test of the minimum inhibitory concentration (MIC) with myriocin and caspofungin used as control, these fungi including *A. fumigatus*, *R. oryzae*, *cryptococcus*, *sporothrix*, *C. krusei* and *C. parapsilosis* (Table 1). Compound **WXP-001** only displayed more outstanding inhibitory activity (MIC = 3.125 μg/mL) against *C. krusei* than caspofungin (MIC = 25 μg/mL), low inhibitory activity against *A. fumigatus*, *R. oryzae* and *sporothrix*. Compound **WXP-002** exhibited moderate activity against all the tested fungi strains with MIC value ranging between 10 and 100 μg/mL, but obviously lower than positive control. Significantly, **WXP-003** showed the highest inhibitory activity against all the tested fungal strains during these three compounds. Anti-fungal activity of **WXP-003** against *C. krusei* and *R oryzae* was superior with MIC value at 0.78 μg/mL and 12.5 μg/mL, while caspofungin only showed MIC value at 25 μg/mL and 100 μg/mL. Similarly, compound **WXP-003** exhibited better activity against *A. fumigatus* and *cryptococcus* (MIC = 3.125 μg/mL, 3.125 μg/mL) than SPT inhibitor myriocin (MIC = 6.25 μg/mL, 6.25 μg/mL).

**Table 1.** Anti-fungal activity of compounds WXP-001∼003.

	MIC (μg/mL)					
Compounds	*A. fumigatus*	*R. oryzae*	*Cryptococcus*	*Sporothrix*	*Candida krusei*	*Candida parapsilosis*
**WXP-001**	50	>100	12.5	100	3.125	12.5
**WXP-002**	25	50	25	50	12.5	100
**WXP-003**	3.125	12.5	3.125	3.125	0.78	1.56
**Myriocin**	6.25	6.25	6.25	1.56	0.195	0.195
**Caspofungin**	0.195	100	0.195	0.39	25	1.56

MIC: minimum inhibitory concentration.

Thus, compound **WXP-003** was selected to test the effect on sphingolipids content in fungi and mouse fibroblast cell line L929, myriocin as positive control and vehicle as negative control (Table 2). The results showed that **WXP-003** and myriocin could reduce the sphingolipids content obviously in *Fusarium graminearum* and *C. albicans*, and the sphingolipids contents treated with **WXP-003** were 454.23 ng/L and 572.97 ng/L, respectively, in *F. graminearum* and *C. albicans*, while vehicle were 553.28 ng/L and 624.40 ng/L, respectively. **WXP-003** had almost no effect on the sphingolipids content in mouse fibroblast cells at the concentration of 1.0 mg/L (sphingolipids content: 832.92 ng/L of WXP-003 and 847.17 ng/L of vehicle). **WXP-003** could significantly reduce sphingolipids content in fungi and no effect on mammal cells.

**Table 2.** Sphingolipids content in two fungal strains and mouse cell line L929 treated with compounds **WXP-003** and myriocin.

	Sphingolipids content (ng/L)		
Compounds	*Fusarium graminearum*^a^	*Candida albicans*a	L929^b^
**WXP-003**	454.23	572.97	832.92
**Myriocin**	428.16	563.34	815.71
Vehicle	553.28	624.40	847.17

^a^The concentration of compounds at 1/2 MIC.

^b^The concentration of compounds at 1.0 mg/L.

In short, **WXP-003** presented the potent anti-fungal activity in this study and then we further explored the molecular dynamics simulation of **WXP-003** with fungi SPT to help us understand the binding mode of **WXP-003** and fungi SPT.

### 3.3. Molecular dynamics simulation

To further investigate the dynamic interaction patterns of SPT/**WXP-003** complex, MD simulation was conducted. First, the stability of the system was evaluated and monitored through the RMSD (root-mean-square deviation) of the backbone atoms. As shown in Supplementary Figure S1, during the last 30 ns, the RMSD values were considerably stable, suggesting that the MD simulation reached equilibrium within the last 30 ns.

**Figure 7.**
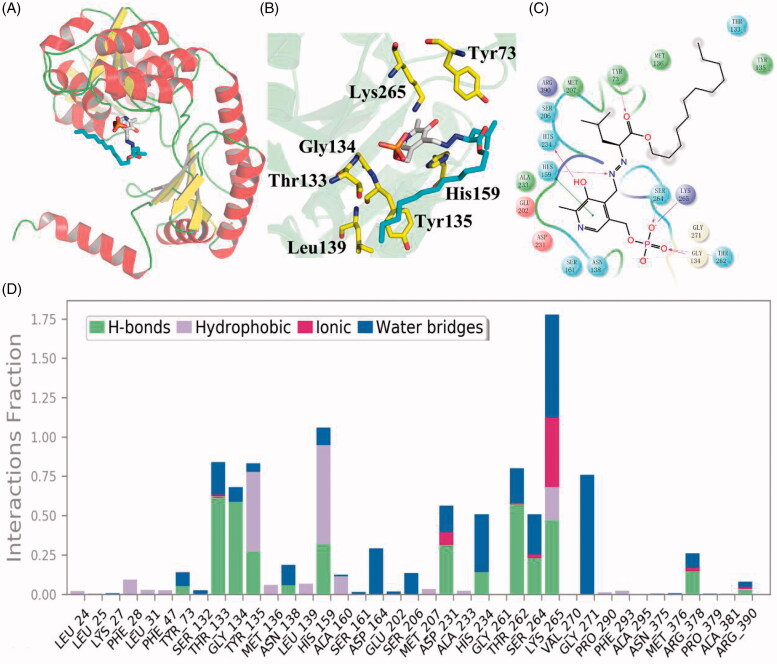
(A) The compound WXP-003 well adapted into the SPT protein. (B) The key residues of the active site of SPT/WXP-003system. (C) 2D presentation of interaction between WXP-003 and SPT. (D) The WXP-003-PLP/residue interaction spectrum of the SPT binding site.

To investigate the binding mechanism of SPT/**WXP-003**, the per-residue contributions in ligand binding were calculated. Figure 7(A) shows that **WXP-003** could well adapt to the binding pocket of SPT, which was similar to the myriocin/SPT complex^11^. **WXP-003** covalent bond to the PLP (pyridoxal 5′-phosphate), and the SPT/**WXP-003** complex also formed the favoured interactions with the key residues within the active site, such as Gly134, His159, Lys265 (Figure 7(B,D))^11^^,^^27^. As shown in Figure 7(C), the carbonyl of **WXP-003** formed a strong hydrogen bond interaction with Tyr73, which may play a key role in the potent bioactivity of **WXP-003**. The key residues–inhibitor interactions are shown in Figure 7(D), in addition to the key contribution discussed above, **WXP-003** could form the strong *Van der Waals* interaction with SPT, especially Tyr135, Leu139, that could make the carbon tail of **WXP-003** more stable in this PLP-SPT system.

### 3.4. Prediction of physio-chemical properties of WXP-003

The correlation between physio-chemical properties of the chemical moieties and the drug pharmacokinetics in the human body was calculated by the Lipinski rule of five. Despite some exception in drug discovery, it is a widely accepted method for preliminary screening of drugs. The results deduced from the molinspiration server are summarised in Table 3. The data disclosed that the molecular weight of **WXP-003** was below 500 and the log *P* values lie in the accepted limits. **WXP-003** had shown 3 hydrogen bond acceptors and 2 hydrogen bond donors.

**Table 3.** Predicted properties of compound **WXP-003.**

Lipinski’s para	
* Lop P*	6.17
* MW*	299.50
* HDA*	3
* HDB*	2
Bioactivity score	
* *TPSA	52.33
* *Molar volume	336.85
* *GPCR ligand	0.18
* *Ion channel modulator	0.23
* *Kinase inhibitor	−0.15
* *Nuclear receptor ligand	−0.07
* *Protease inhibitor	0.40
* *Enzyme inhibitor	0.27

Calculated from online server http://www.molinspiration.com/cgi-bin/properties. HDA: number of hydrogen bond acceptors; HBD: number of hydrogen bond donors; MW: molecular weight; Log*P*: logarithm of partition coefficient between n-octanol and water (miLog*P*); TPSA: topological polar surface area. A molecule having bioactivity score more than 0.00 is likely to exhibit considerable biological activity, while value −0.50 to 0.00 are expected to be moderately active and if the score is less than −0.50 it is presumed to be inactive.

## 4. Conclusion

In summary, we have constructed homology models of human serine palmitoyltransferase (SPT) for virtual screening. A logical docking-based virtual screening approach was employed to identify novel fungi SPT inhibitors while except human SPT inhibitors. In our study, **WXP-003** was screened through *in silico* and in MIC assays and was synthesised. **WXP-003** showed strong inhibitory activity against the tested fungal strains. **WXP-003** could significantly reduce sphingolipids content in fungi and no effect on mouse fibroblast cell L929. Meanwhile, molecular dynamics simulation was conducted to investigate the dynamic interaction patterns of SPT/**WXP-003** complex, and then the SPT/**WXP-003** complex formed the favoured interactions with the key residues within the active site. The prediction of physio-chemical properties provided further evidence that **WXP-003** may be a promising anti-fungal agent. This work offers a new potential approach based on discovering selective fungi SPT inhibitor.

## Supplementary Material

Supplemental Material
