# Supplementary material for: Identification of a novel SPT inhibitor WXP-003 by docking-based virtual screening and investigation of its anti-fungi effect
Source: J Enzyme Inhib Med Chem. 2021 Jun 21;36(1):1007–15. doi: 10.1080/14756366.2021.1915301 (PMC8218698; doi:10.1080/14756366.2021.1915301)

Identification of a novel SPT inhibitor WXP-003 by docking-based virtual screening and investigation of its anti-fungi effect

Xin Wang [⊥ a], Xin Yang[a], Xin Sun[a], Yi Qian[a], Mengyao Fan[a], Zhehao Zhang[a], Kaiyuan Deng[a], Zaixiang Lou[c], Zejun Pei [*⊥a] and Jingyu Zhu [*b]

[a]*The Affiliated Wuxi No.2 People's Hospital of Nanjing Medical University, 68 Zhongshan Road, Wuxi 214002, China*

[b] *School of Pharmaceutical Sciences, Jiangnan University, 1800 Lihu Road, Wuxi 214122, China*

[c] *School of Food Science and Technology, Jiangnan University, 1800 Lihu Road, Wuxi 214122, China*

**CONTACT*Zejun Pei (pei-zj@njmu.edu.cn). The Affiliated Wuxi No.2 People's Hospital of Nanjing Medical University, 68 Zhongshan Road, Wuxi 214002, China; JingyuZhu (jingyuzhu@jiangnan.edu.cn). Jiangnan University, 1800 Lihu Road, Wuxi 214122, China. These authors contribute equally.*

Author Contributions: [#]Xin Wang and Zejun Pei are contributed equally to this work.

Figure S1.The root-mean-square deviation (RMSDs) of the backbone atoms of SPT/WXP-003 complex... 3

Table S1. Inhibitory activity of compounds WXP-001~005 against diversity fungal strains... 4

Copy of 1H- Spectra and LC-MS Spectra for Compound WXP-001..................... 5

Copy of 1H- Spectra and LC-MS Spectra for Compound WXP-002......................6

Copy of 1H- Spectra and LC-MS Spectra for Compound WXP-003......................7

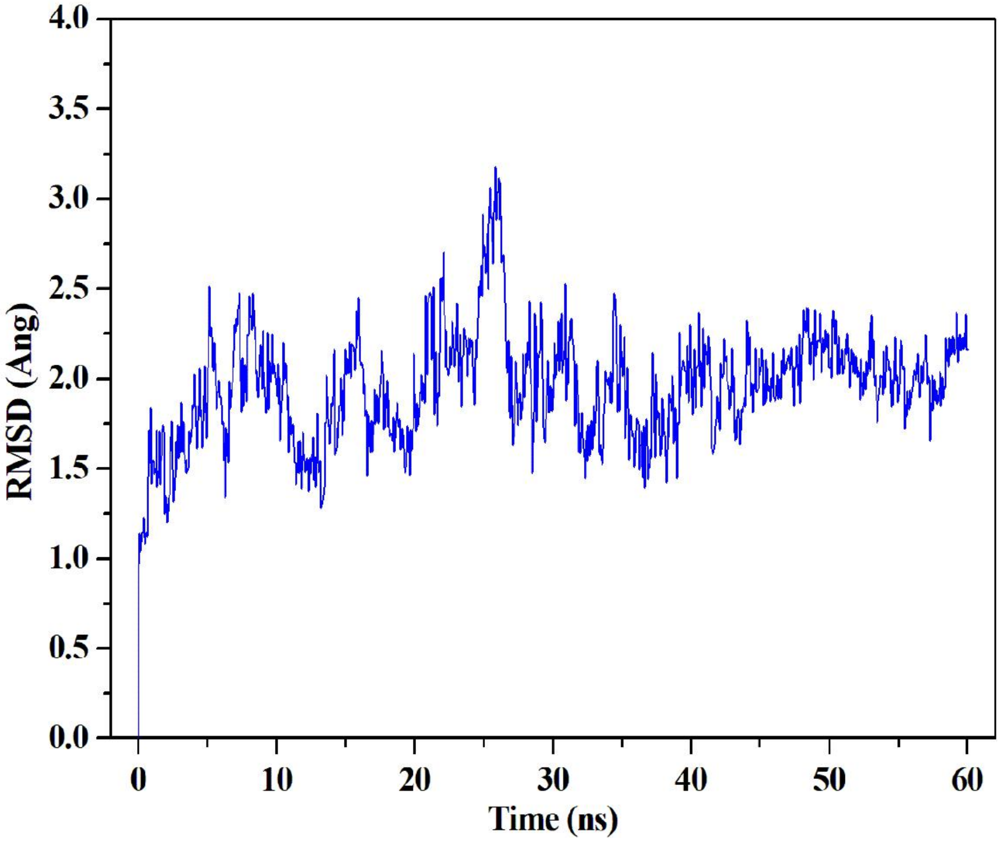

Figure S1. The root-mean-square deviation (RMSDs) of the backbone atoms of SPT/**WXP-003** complex.

Table S1. Inhibitory activity of compounds **WXP-001~005** against diversity fungal strains.

Compds	Standard fungal strains															Clinical drug-resistance fungal strains								
	candida tropicalis 186815			*candida parapsilosis* 336515			*candida glabrata* 337348			*candida krusei* 185429			*candida lusitaniae* 340928			*candida tropicalis* 191529			*candida tropicalis* 191327			*candida parapsilosis* 191344		
	C_1	C_2	C_3	C_1	C_2	C_3	C_1	C_2	C_3	C_1	C_2	C_3	C_1	C_2	C_3	C_1	C_2	C_3	C_1	C_2	C_3	C_1	C_2	C_3
**WXP-001**	–	–	+	–	–	+	–	–	+	–	–	+	–	–	+	–	–	+	–	–	+	–	–	+
**WXP-002**	–	–	+	–	–	+	–	–	+	–	–	+	–	–	+	–	–	+	–	–	+	–	–	+
**WXP-003**	–	–	–	–	–	–	–	–	–	–	–	–	–	–	–	–	–	–	–	–	–	–	–	–
**WXP-004**		+	+	–	–	+	±	+	+	+	+	+	–	–	±	+	+	+	+	+	+	–	–	±
**WXP-005**	–	+	+	+	+	+	+	+	+	–	+	+	–	+	+	+	+	+	–	+	+	+	+	+

C_1: the concentration of compounds at 100 μg/mL
C_2: the concentration of compounds at 50 μg/mL
C_3: the concentration of compounds at 10 μg/mL
"+": means obvious growth of fungi
"±": means less growth of fungi
"–": means no growth of fungi

Copy of 1H- Spectra for Compound WXP-001

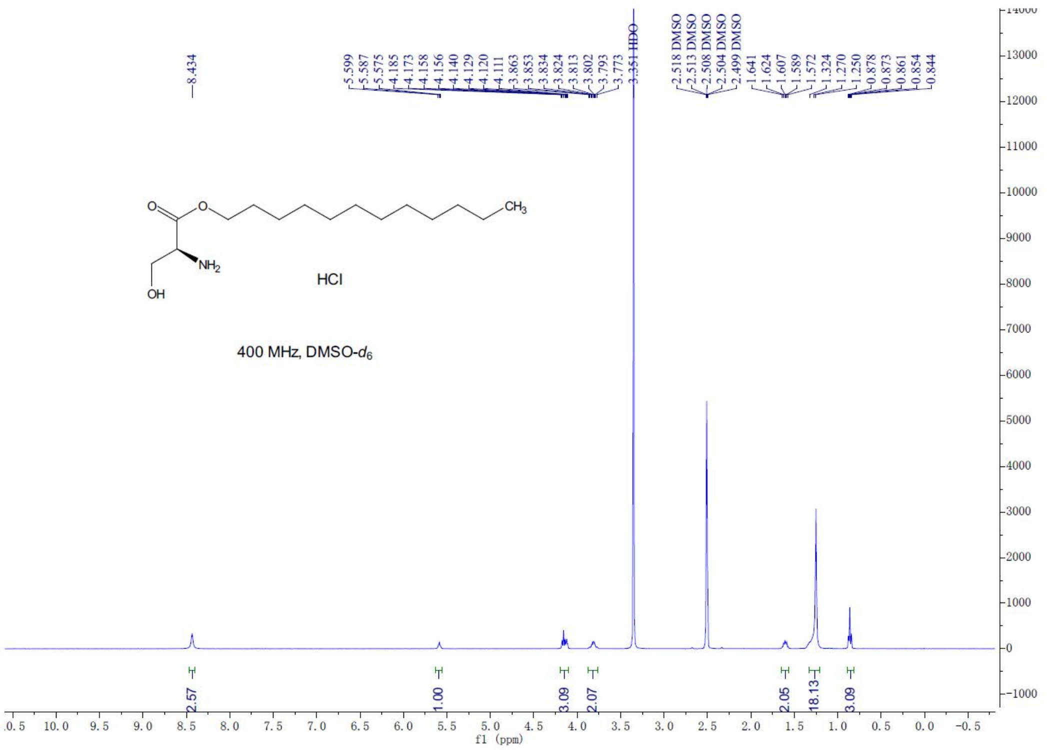

Copy of LC-MS Spectra for Compound WXP-001

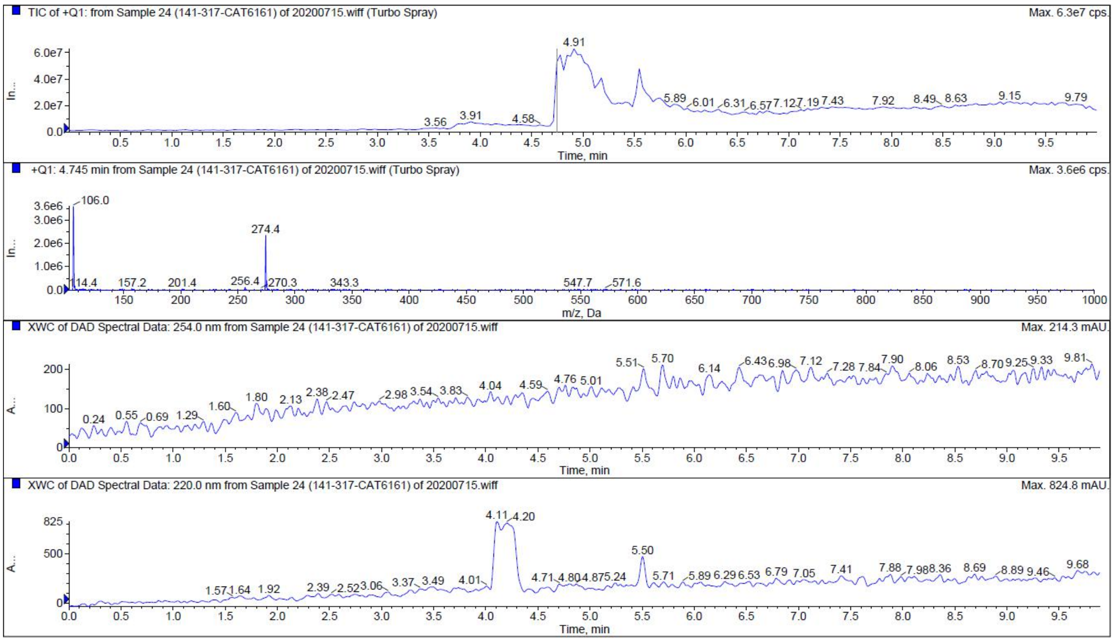

Copy of 1H- Spectra for Compound WXP-002

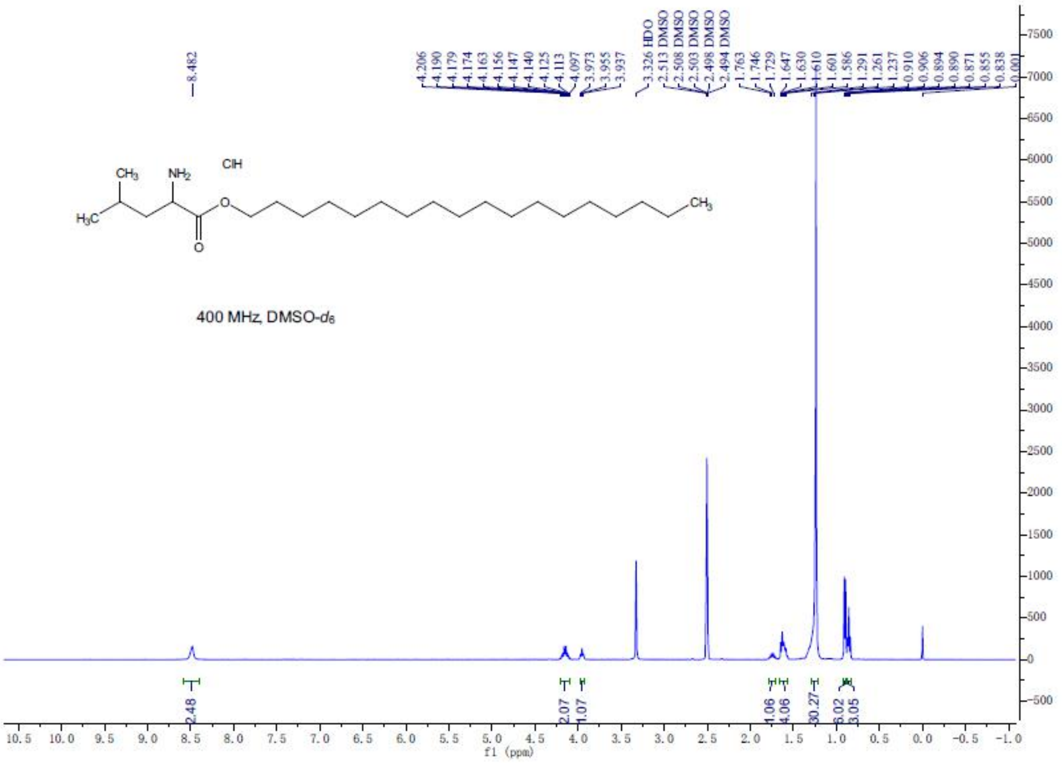

Copy of LC-MS Spectra for Compound WXP-002

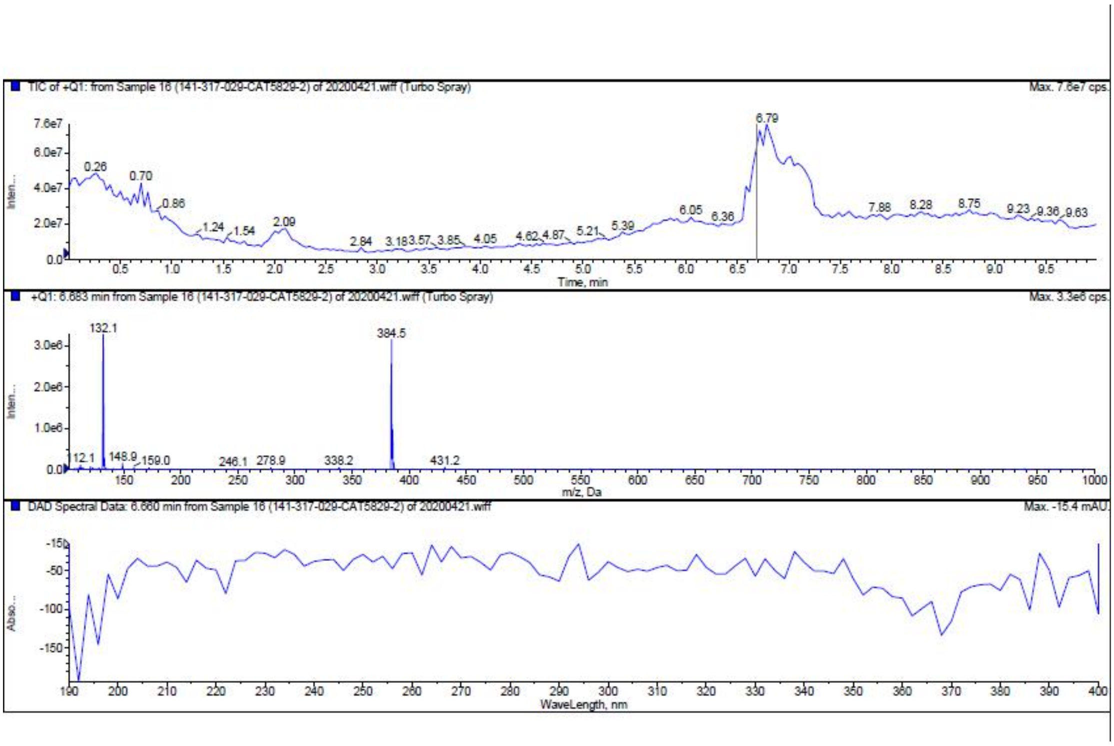

Copy of 1H- Spectra for Compound WXP-003

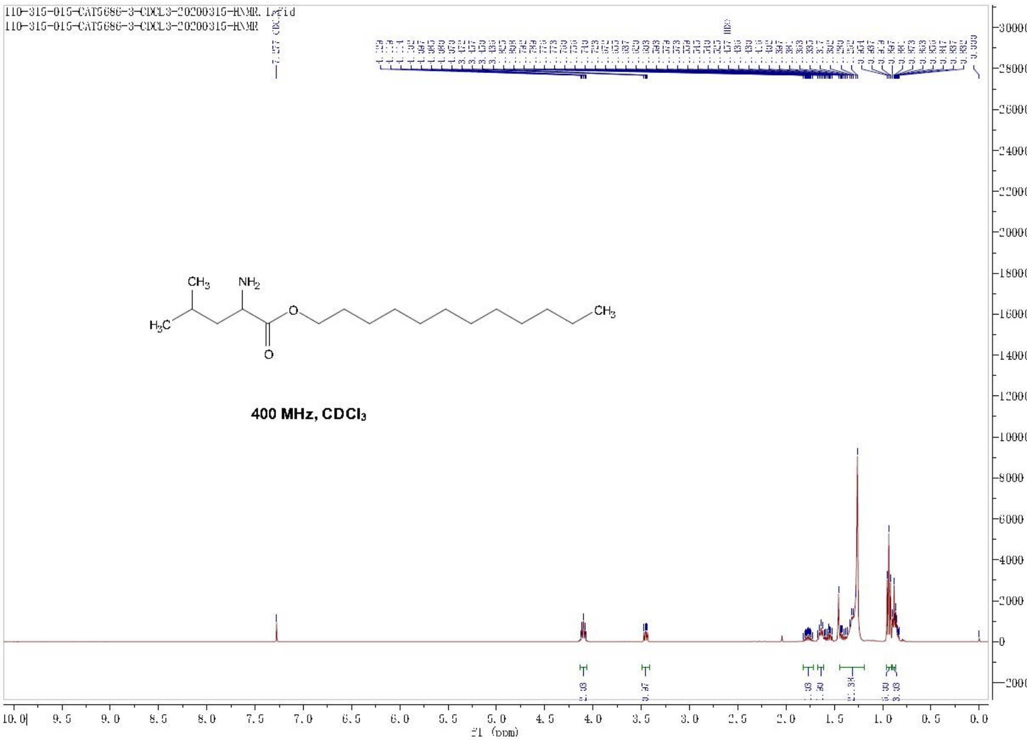

Copy of LC-MS Spectra for Compound WXP-003

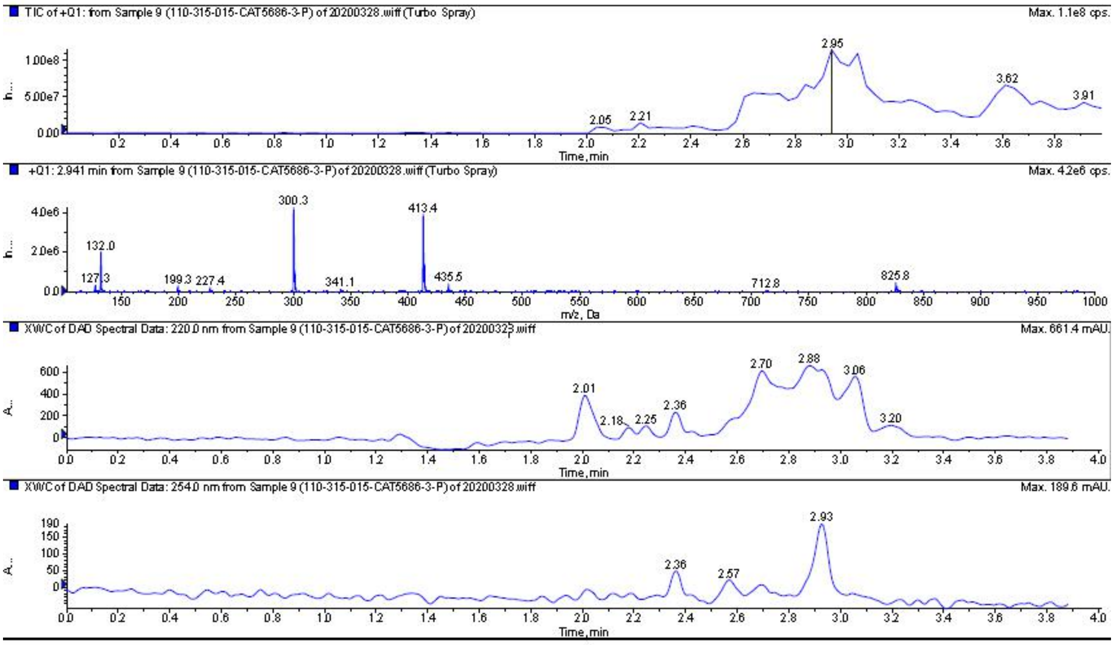

Supplement: Supplemental Material [file IENZ_A_1915301_SM4927.pdf]
